# Cryopreserved Rat Thyroid Autotransplantation in the Treatment of Postoperative Hypothyroidism

**DOI:** 10.3389/fendo.2021.625173

**Published:** 2021-05-17

**Authors:** Marcel Vasconcellos, Amabile Maran Carra, Olavo Borges Franco, Wagner Baetas-da-Cruz, Manoel Luiz Ferreira, Paulo Cesar Silva, Sergio Augusto Lopes de Souza, Leandro Miranda-Alves, Denise Pires de Carvalho, Alberto Schanaider

**Affiliations:** ^1^ Post Graduate Program in Surgical Sciences, Department of Surgery, School of Medicine, Federal University of Rio de Janeiro, Rio de Janeiro, Brazil; ^2^ School of Medicine, Federal University of Rio de Janeiro, Rio de Janeiro, Brazil; ^3^ Post Graduate Program in Surgical Sciences, Center of Experimental Surgery, Department of Surgery, School of Medicine, Federal University of Rio de Janeiro, Rio de Janeiro, Brazil; ^4^ Center of Experimental Surgery, Department of Surgery, School of Medicine, Federal University of Rio de Janeiro, Rio de Janeiro, Brazil; ^5^ Department of Radiology, School of Medicine, Federal University of Rio de Janeiro, Rio de Janeiro, Brazil; ^6^ Institute of Biomedical Sciences, Federal University of Rio de Janeiro, Rio de Janeiro, Brazil; ^7^ Institute of Biophysics Carlos Chagas Filho, Federal University of Rio de Janeiro, Rio de Janeiro, Brazil

**Keywords:** thyroidectomy, cryopreservation, autologous transplantation, rats, endocrinology

## Abstract

To verify the viability and functionality of cryopreserved thyroid autotransplantation in rats who underwent total thyroidectomy in the treatment of postoperative hypothyroidism. Thirty-two Wistar rats were randomly assigned into groups (G) with eight animals each: control (CG); simulation (SG); hypothyroidism (HTG) and transplanted (TG). At the beginning and in the 13th week of the experiment, serum levels of total T3, free T4, TSH and calcium were determined. In both the first and 14th weeks, scintigraphic examinations, 99m-Tc pertechnetate radioisotope biodistribution and histopathology were performed. In the 14th week, the expression of proliferating cell nuclear antigen (PCNA) and cellular apoptosis (caspase-3) were also evaluated. In the 13th week, the transplanted animals had normal serum levels of total T3 and free T4. TSH levels showed a tendency towards normality. In the 14th week, scintigraphic exams displayed graft isotopic uptake in all animals in the TG group. Histological examinations 13 weeks after transplantation showed the viability and functionality of thyroid follicles. PCNA revealed significant immunoreactivity of the graft (*p* < 0.001) when the TG was compared to the CG. There was no difference between CG and TG considering the expression of activated caspase-3. The experimental study confirmed the viability and functionality of thyroid autotransplantation implanted in skeletal muscle with evidence of cell proliferation without cellular apoptosis. This surgical strategy was effective in the treatment of postoperative hypothyroidism.

## Introduction

Total or subtotal thyroidectomy can generate a clinical condition that requires permanent hormonal supplementation compromising the patient’s quality of life. Postoperative hypothyroidism may arise from the treatment of toxic diffuse goiter (Basedow-Graves’ disease), toxic or non-toxic multinodular goiter (endemic goiter), or cancer, or because of iatrogenic procedures, for more extensive resections than necessary (1).

The authors conducted a survey, searching full access papers and abstracts indexed in the MEDLINE/PubMed (National Institutes of Health), Cochrane Database of Systematic Reviews and SciELO (Scientific Electronic Library Online) databases using the following keywords (using “AND” or “OR”): autotransplantation, thyroid, total thyroidectomy, and thyroid autograft. Ultimately, 32 papers were found, but different laboratory animal species were used (rats, dogs, guinea pigs and rabbits), some grafts were transplanted immediately during the surgical procedure while several others were cryopreserved and transplanted weeks before. The postoperative intervals for transplantation were also different (1–7). Although autologous thyroid transplantation studies suggested some encouraging results, the surgical strategy have not been consistent.

To determine the clinical applicability of thyroid autotransplantation, the authors developed an experimental study to test the viability of autologous transplantation of healthy thyroid glandular tissue after a period of cryopreservation. This investigation with translational impact may improve the knowledge and support a surgical alternative for the treatment of postoperative hypothyroidism, avoiding permanent thyroid hormone replacement.

## Materials and Methods

This study was approved by the Ethics Committee on the Use of Animals of the Federal University of Rio de Janeiro (No.111/2017), in accordance with the guidelines of the International Care and Use Committee of the National Institute of Health, and Guide for the Care and Use of Laboratory Animals.

For the development of this research, 32 albino rats (*Rattus norvegicus*), Wistar lineage males, with an average age of 3 months and a mean weight of 300 ± 20 gm, were used. The rats were housed in environmentally controlled conditions and fed with standardized food and water *ad libitum.*

Wistar rats were randomly assigned into groups (n = 8, each one): Control group (CG), without surgical procedure; Simulation group (SG), only with surgical access (sham surgery); Hypothyroidism group (HTG), total thyroidectomy; and the Transplanted group (TG), total thyroidectomy with cryopreservation of the thyroid gland for 7 days, followed by grafting of one thyroid lobe in a muscular layer of the hind leg.

### Operative Procedures for Total Thyroidectomy

The anesthetic procedure comprises a solution of 10% ketamine hydrochloride (100 mg/kg) plus 2% xylazine hydrochloride (10 mg/kg), intraperitoneally. An 8-inch longitudinal skin and platysma muscle incisions along the cranial-caudal midline of the ventral cervical region followed by separation of the sternocleidomastoid and sternohyoid muscles were done. A binocular microscope with 10X magnification to perform a meticulous dissection of the thyroid gland preserving the parathyroid glands, recurrent laryngeal nerves and vascular structures was used. The parathyroid glands were detached from the thyroid with gentle movements using a cotton swab-type flexible rod moistened with saline solution and when necessary, a microsurgical scissors helped in the dissection. To close the surgical wound, simple stitches of both muscular and skin layers, respectively with 4-0 polyglycolic acid (Vicryl^®^, Ethicon, Brazil) and 3-0 nylon (Mononylon^®^, Ethicon, Brazil), were performed. After the total thyroidectomy a postoperative analgesia was administered (Tramadol hydrochloride,1 mg/kg, i.p. and oral ibuprofen 30 mg/kg).

### Cryopreservation of the Thyroid Gland

The entire left lobe of the thyroid gland was kept in a 1x sterile PBS solution (pH 7.4). In a laminar flow chamber, a solution containing 43% RPMI 1640 (Cultilab, SP, BR), 50% bovine fetal serum (Sigma, Mo, USA), and 7% DMSO (dimethylsulfoxide) placed in a cryotube and stored in a freezer for 1 hour, followed by storage for seven days in liquid nitrogen. The defrosting used a water bath until the sample reached room temperature, and two washes were performed with sterile PBS 0.001 M, pH = 7.4.

### Operative Technique of Tissue Grafting

Each animal served as its own donor (autologous transplantation), and the entire left lobe, weighting on average 6.12 mg, was implanted in the biceps femoris muscle of the right hind leg at the middle third of the thigh surgically.

### Determination of Serum Concentrations of Total T3 (Triiodothyronine), FT4 (Free Thyroxine), TSH (Thyrotrophic Hormone) and Total Calcium

The concentrations of total T3 (nmol/L), free T4 (pmol/L) and TSH (mUI/L) were determined by the chemiluminescence method (LumiQuest^®^, Lagoa Santa, MG, BR).Total calcium (mmol/L) was determined by the automated kinetic method (LabTest^®^, Lagoa Santa, MG, BR).

### Scintigraphic Examinations

In the 14th week, after injection with a 0.35 µCi dose, the pertechnetate-99mTc uptake of the transplanted gland (TG) in the biceps femoris contralateral muscle as well as *ex vivo* was studied. Biodistribution analysis of pertechnetate-99mTc in the CG and TG groups were also determined. Scintigraphic examinations were performed by simple photon emission tomography and image acquisition and processing in Digital Imaging and Communications in Medicine format.

### Histological and Immunohistochemistry Examinations

The samples of the transplanted left lobe were stained with hematoxylin and eosin (H&E) and analyzed under optical microscopy (E200 Nikon, SP, BR) at magnifications of 200 and 400 times.

Antigen recovery of PCNA was performed with 0.01 M citric acid buffer/pH = 6.0 and microwave heating. Antigen recovery of caspase-3 was performed with A+B buffer (sodium citrate + citric acid). Inactivation of endogenous peroxidase was performed with hydrogen peroxide. The sections were incubated with anti-PCNA primary antibody (anti-PCNA F-2 # E0713 monoclonal mouse, Santa Cruz Biotechnology, CA, USA) or anti-caspase-3 (anti-casp 3, p11 monoclonal mouse, Cat # SC-271759 Santa Cruz Biotechnology, CA, USA), in 1/100 dilutions in a darkroom, overnight, at 4 °C. The positivity pattern was based on calculating the percentage of immunoreactivity in at least 500 cells. For each of the four slides of each marker, positive follicular cell counts were performed, with x200 magnification.

### Statistical Analyses

**Figure 1:**
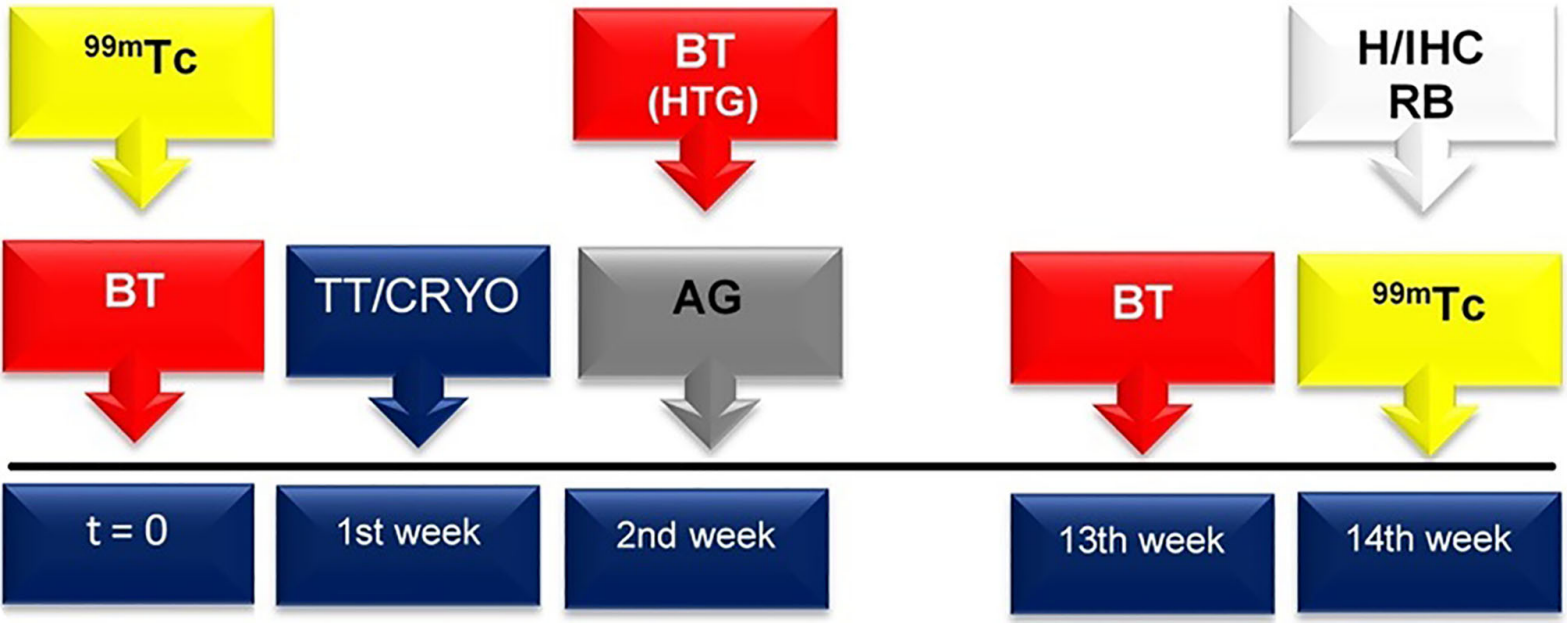
At both t = 0 and 13 weeks, biochemical tests (BT), including serum levels of total T3, free T4 and TSH, and total calcium, were performed for all groups (red box with arrow). In the HTG, the thyroid hormone and calcium serum levels were also determined one week after thyroidectomy (that is, in the 2nd week after the beginning of the experiment). Total thyroidectomy (TT) and cryopreservation (CRYO) of the thyroid gland occurred in the 1st week (blue box with arrow). In the 2nd week, the autologous graft (AG) was implanted in the TG (grey box with arrow). In both the t = 0 and 14th weeks, scintigraphic examination (99mTc) was accomplished (yellow box with arrow). In the 14th week, histological evaluation (H), immunohistochemistry analysis (IHC) and radioisotope biodistribution (RB) were performed (white box with arrow).

The Shapiro-Wilk normality test, analysis of variance (ANOVA), and Tukey test were used. In all tests, a 95% confidence interval (CI = 95%) and 5% statistical significance (*p* < 0.05) were used. The analyses were performed using the statistical program SPSS version 22.0 (Belmont, CA, USA). The timeline of the procedures are summarized in **Figure 1**.

## Results

### Serum Concentrations of Total T3, Free T4, TSH and Total Calcium

One week after thyroidectomy, the HTG showed a significant reduction in free T4 and elevated TSH serum levels when compared with those in the CG and SG groups (*p* < 0.05). There were no significant differences among the CG, SG and HTG groups for total T3 (*p* = 0.23).

**Figure 2:**
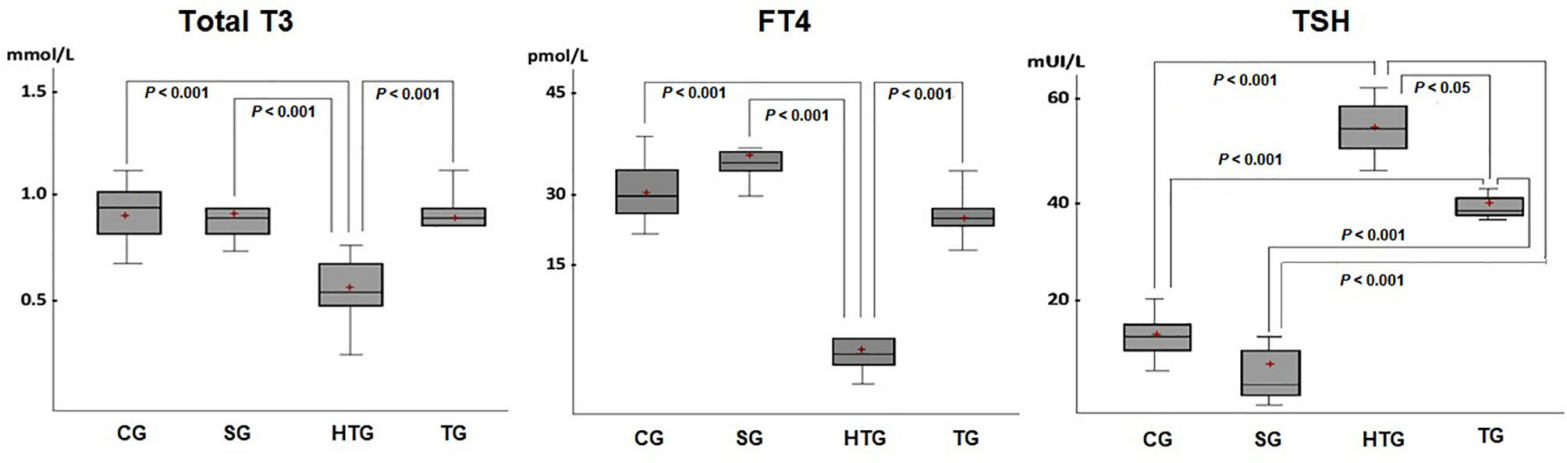
There was a decrease in total T3 serum level in the HTG compared with the results of the other groups (*p* < 0.001), which showed no significant differences among them. A significant reduction in the free T4 serum level was also observed in the HTG compared to the TG, CG and SG. An increase in TSH levels was found in both the HTG and TG, but with higher values in the former TG (*p* < 0.05). Horizontal bars represent medians, boxes represent the 25th and 75th percentiles, and vertical bars represent ranges. The level of significance was set at *p* < 0.05.

In the 13th week, a reduction in the concentration of total T3 in the HTG group compared to the CF, SG and TG groups was observed (*p* < 0.001) (**Figure 2**). There was no significant difference in total calcium among the groups.

### Scintigraphic Examinations

**Figure 3:**
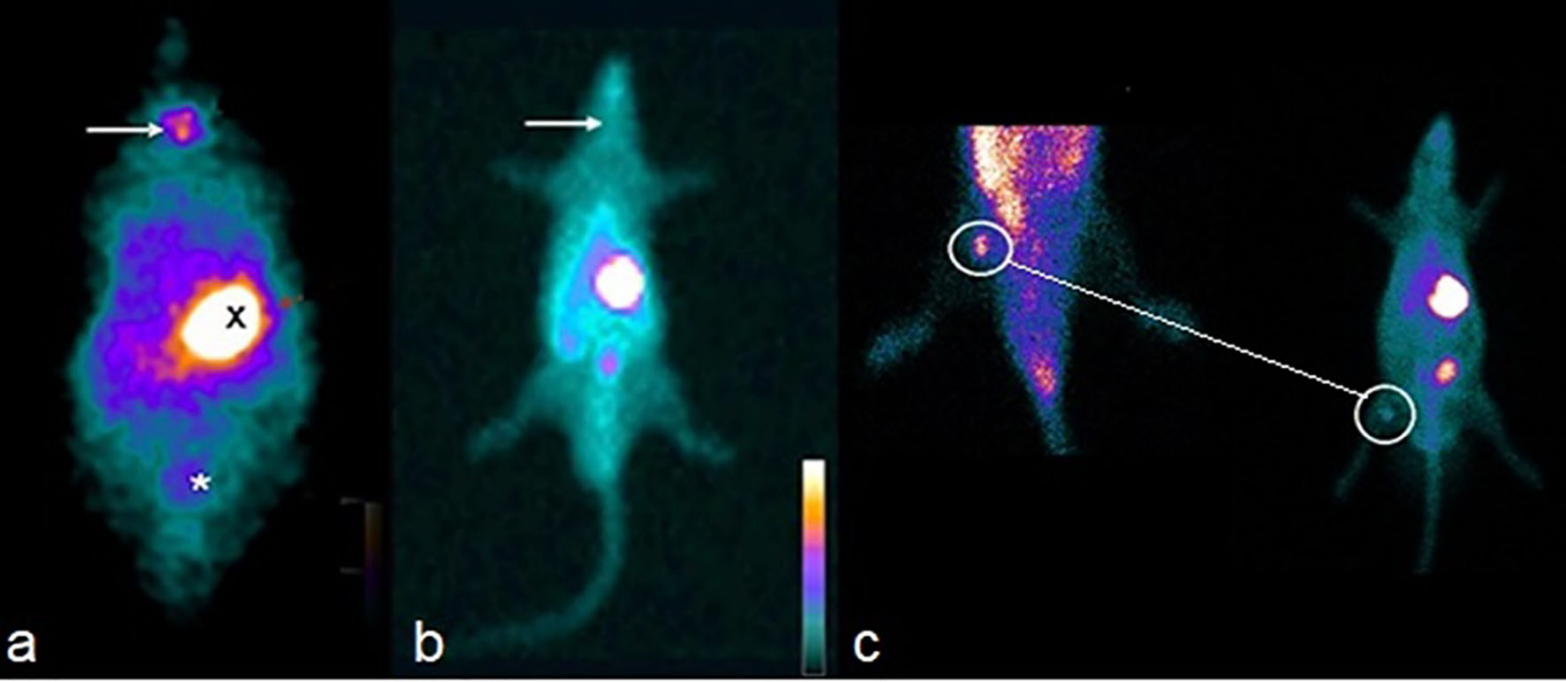
**(A)** Whole-body scan showing isotope capture of the radioisotope in the thyroid gland topography (arrow), epigastric region (X) and bladder (*) of the CG. **(B)** Absence of isotope uptake of the radiotracer in the cervical topography (arrow) of the HTG. **(C)** Isotope uptake of the technetium by the graft (surrounded by circles), confirming its viability 12 weeks after the implant in TG. In the small image of the rat (at right), it is also seen that there is an absence of isotope uptake in the cervical location of the TG.

In both the t = 0 and 14th weeks, whole-body scan scintigraphy performed in CG and SG groups showed uptake in thyroid tissue and elimination by the digestive and renal systems in a normal pattern (**Figures 3A, B**). In the 14th week, the tissue was viable (**Figure 3C**).

### Analysis of the Radioisotope Biodistribution Pertechnetate-99mTc

The uptake rate of the thyroid graft in the right biceps femoral muscle in the TG was higher than that found in the animals’ own contralateral muscle (*p* < 0.001). In the other organs/tissues studied, no significant differences were found among all groups (*p* > 0.05).

### Histopathological Analysis

**Figure 4:**
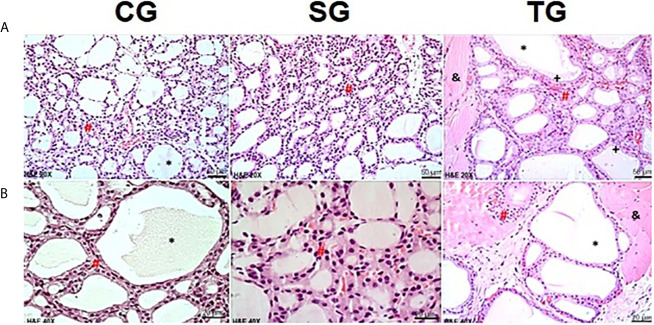
Photomicrographs of the thyroid gland in CG, SG and TG in the 14th week. In the CG and SG, it was possible to observe intact thyroid follicles, consisting of a simple epithelial layer (#), whose content is filled with colloid (*). Images of the transplanted animals (TG) showed follicles containing numerous vesicles of endocytosis (+). In TG animals, the presence of muscle tissue was observed between follicular cells (&). (H&E, magnification of x200 and x400). Scale bars = 50 for **(A)** Scale bars= 10 µm for **(B)**.

In the 14th week, HTG showed not only thyroid follicles coated with simple columnar epithelium but also the presence of stored hormones with colloid patterns similar to those seen in the CG and SG groups. Similar findings were found in the TG group. However, the follicles contained numerous endocytosis vesicles (**Figure 4**).

### Immunohistochemistry Analyses

**Figure 5:**
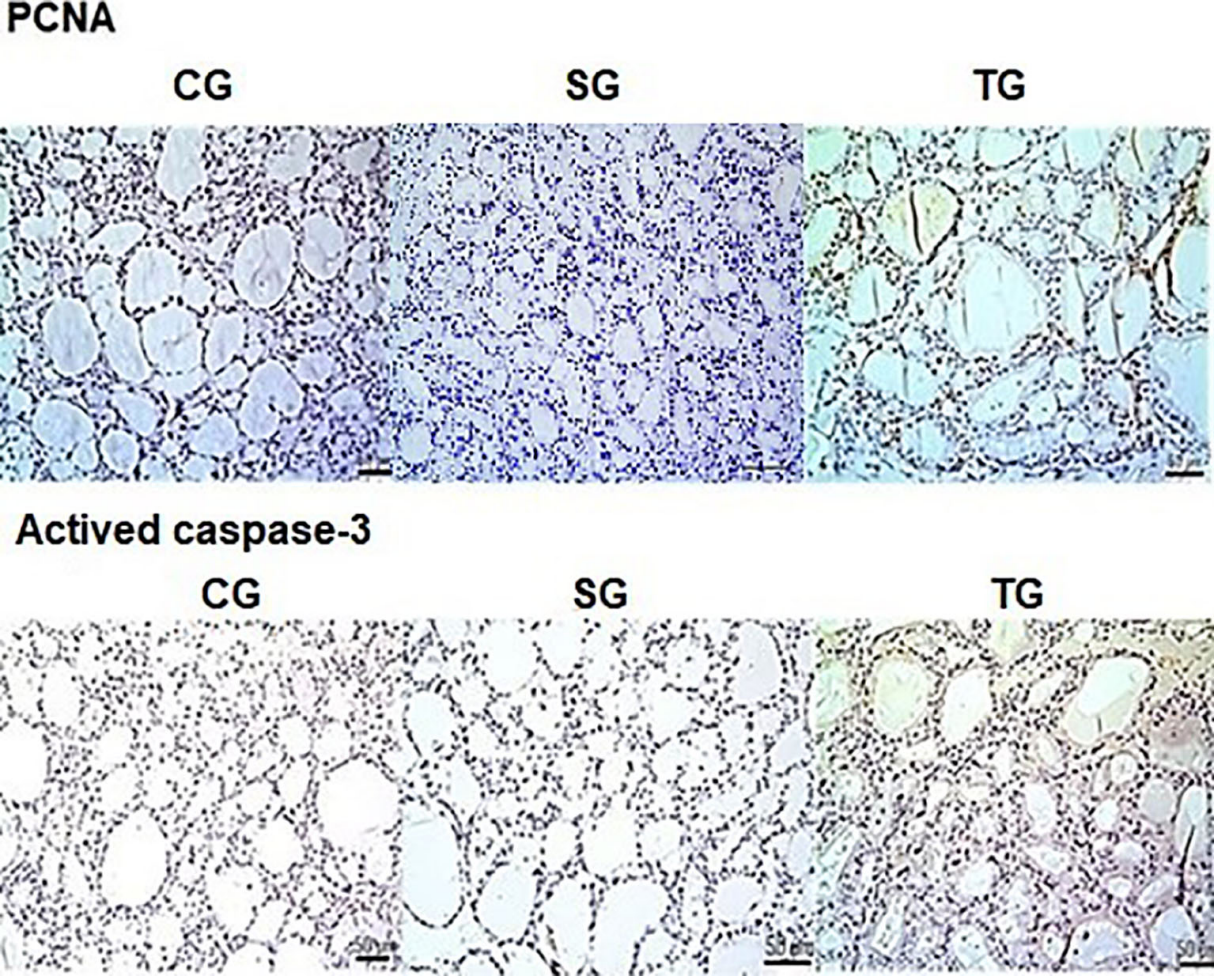
There was a statistically significant increase (*p* < 0.001) in the expression of PCNA in the TG compared to the CG (brown color marking). No differences were observed caspase-3 antibody immunoreactivity between both groups. Scale bars: 50 µm.

The reaction with monoclonal anti-PCNA antibodies identified a significant elevation (*p* < 0.001) in the cell proliferation marker in the TG group compared to the CG group. No difference between TG and CG for caspase-3 monoclonal antibody immunoreactivity was identified (**Figure 5**).

## Discussion

The efficacy of a permanent hormone replacement in the presence of postoperative hypothyroidism may be limited by the patient’s lack of adherence to treatment, inadequate dose administration, malabsorption, among other reasons. In addition, free T4 treatment for a prolonged period may trigger deleterious side effects in patients with cardiovascular diseases, diabetes, using anticoagulant drugs, and women in the postmenopausal period (1, 8, 9). A multicenter study found that approximately 50% of patients undergoing such treatment had thyroid stimulating hormone (TSH) levels within the normal range (10).

Experimental and clinical research on thyroid gland autotransplantation is rare, and the methodology used varies considerably, without uniformity. Thus, it is difficult to perform a critical comparative analysis. Various species (dogs, mice, rabbits, rats, pigs and hamster) as well as samples with a frequent lack of identification of the number of animals have been reported (1, 3, 8). Furthermore, two relevant surgical techniques (immediate transplantation *versus* cryopreservation), in addition to several different locations of the implanted tissue, have been found. Finally, different types of grafts (culture or emulsion *versus* thyroid fragments) and postoperative follow-up times have been used (1, 8, 11–17).

The anatomy, histology and physiology of the rat thyroid are quite similar to those found in humans and allowed us to develop a reproducible experimental model capable of adding a translational applicability to autologous cryopreserved thyroid transplantation.

Choosing the ideal location for thyroid transplantation has to take into account several factors, such as a less invasive procedure, easy access and a low-risk incision. Hence, our choice was based on experimental and clinical studies whose heterotopic grafts were successfully implanted in skeletal muscle tissue with greater clinical applicability (12, 15, 17).

In most of the experimental and clinical studies obtained from the database searches, total thyroidectomy was almost exclusively performed (18, 19). In fact, partial glandular resection may add a confusing factor since the functional effects of the remaining thyroid could overlap and mask those from the graft itself.

Cryopreservation is a well-established technique and has been used more often with other kinds of tissues (5, 20). In the process of cryopreservation of the thyroid tissue, the technique of slow freezing was chosen (rate of -1°C per minute) because it gradually exposes the cells to low temperatures, preventing the formation of ice crystals in the cell cytoplasm (3, 21, 22). However, the cryopreservation process of the thyroid gland has been used in only three studies in humans, ranging from 4 months to 3.5 years (18, 23, 24). Shimizu et al. reported a thyroid graft implanted in muscle that remained functional in 3 of 4 patients for 2 to 7 years later (18). Likewise, Gál et al. reported glandular functionality 30 days after cryopreserved thyroid transplantation in dogs (2).

In both the second and 13th weeks, a comparison of HTG to CG and SG groups showed a reduction in free T4 and an increase in TSH (*p* < 0.05) serum levels, demonstrating the effectiveness of the surgical procedure to cause hypothyroidism. It is noteworthy that the postoperative time of 13 weeks chosen in the experimental model in *Rattus norvegicus* would be equivalent to 7 months in humans, considering the analogy with the life expectancy of both species. In clinical-surgical studies, this interval of time is sufficient for the development of postoperative hypothyroidism. In the 13th week, the results of T4 and T3 levels of the CG and the SG groups compared to the TG group showed no significant differences. There was also a reduction in the serum levels of TSH in the TG group compared to the HTG group (*p* < 0.001), with a tendency to normality. Similar results were described in a study with *anima nobile* after cryopreservation (18) and in other animal species (2, 12). Monhsen et al. in 2017, reported the postoperative evolution of 40 patients with autologous thyroid emulsion transplantation, and 12 months later, the TSH remained high, despite T3 and free T4 levels remaining in the normal range (15). Okamoto et al. studied 5 patients with Graves’ disease who underwent subtotal thyroidectomy with immediate thyroid autotransplantation in the sternocleidomastoid muscle. In all patients, followed from 2 to 7 years, there was normalization of T3 and free T4, but with mildly elevated TSH serum levels, in three patients. In 80% of the patients, the radioisotope administered was taken up by the graft (19). Most transplanted human patients maintained high concentrations of TSH for several months. Nevertheless, over time (up to one year), there was a tendency towards the euthyroid state, similar to our findings.

The full body scan of the HTG animals in the 14th week confirmed the absence of radioisotope uptake in the location of the thyroid gland. The radioisotope used in this study has both satisfactory sensitivity and high positive predictive value (PPV), 79% and 100%, respectively, for screening remaining tissue after total thyroidectomy (14). In the 14th week, all animals in the TG group had isotope uptake at the site of the graft (right thigh of the hind limb). This result exceeded those reported by Dobrinja and colleagues in rats, who cited a successful rate of 70% at 4 week after the graft had been implanted in the rectus abdominis muscle (25). Roy et al. described 6 patients with non-toxic multinodular goiter who underwent immediate autologous thyroid transplantation who remained euthyroid at 6 months postoperatively, but only 45% had radioisotope uptake (26). Mohsen et al. after applying thyroid tissue emulsion to the thigh muscles at the same surgical time of a total thyroidectomy, reported 99mTc radioisotope uptake in all grafts (15). Indeed, our results demonstrate the viability of functional thyroid tissue transplanted after cryopreservation.

We identified only one study with a graft that originated from cryopreserved tissue, but with no histopathological description (23). In our study, in the 14th week, the TG group had a graft with thyroid architecture similar to the control and sham groups. The thyroid follicles were filled by colloids, and there were vesicles of endocytosis, indicating hormonal activity. These histological findings were consistent with the findings of Yoshizaki et al. and Dobrinja et al., who performed transplantation with tissue preserved in culture medium (25, 27). Also supporting our results, Karaman et al. at day 60, described 100% functional thyroid follicles in all grafts of animals (hamsters) who underwent immediate transplantation (14).

We used molecular markers in the present study to assess cellular viability. The highest positivity of the PCNA immunomarker (the proliferating cell nuclear antigen) in the TG group suggests active DNA replication of the transplanted thyroid tissue. However, it is still unknown how long this activity would persist and the precise molecular mechanisms involved in this process. In the current study, the reaction of samples with cleaved caspase-3 monoclonal antibodies in the 14th week did not identify cellular apoptosis markers in grafts (TG), showing a similar result to those animals whose thyroid cells remained in physiological conditions (CG).

The surgical procedures used for the treatment of thyroid disease vary considerably. Subtotal thyroidectomy or lobectomy have been performed for less aggressive or low-risk variants of follicular carcinoma, but several groups worldwide have always recommended total thyroidectomy because of its multicentricity and unpredictable prognosis (28). Total thyroidectomy performed in the presence of malignant neoplasms, or even multinodular toxic goiter, will certainly require postoperative hormone replacement. An extensive bilateral subtotal thyroidectomy may be indicated for non-toxic goiters (endemic with iodine deficiency) or, in most cases, Basedow-Graves’ disease (for instance, diffuse toxic goiters, focal toxic goiters with compressive symptoms, failure of treatment with antithyroid drugs in pregnant women, allergy to antithyroid drugs or refusal of 131 iodine treatment). In such circumstances, hypothyroidism is common (29).

Another major challenge for the surgeon is the subjective visual intraoperative quantification of the remaining glandular size required to maintain the euthyroid state. The amount of thyroid tissue transplanted in our study corresponded to 44% of the absolute weight of the murine gland. After 12 months of observing human grafts with a weight equal to or larger than 10 g, which means approximately 40 - 66% of the average absolute weight of the human gland, Mohsen et al. concluded that such features could ensure the best functional results. In more conservative operations, such as partial or subtotal thyroidectomies, the recurrence of the disease may occur, ranging from 5% to 20% (15). Lin et al. who studied 415 patients with Graves’ disease after surgery, reported nearly 50% hypothyroidism (29).

There are some limitations to our study. The experimental study was carried out in healthy animals without thyroid diseases. The freezing time with hypothermic storage of the thyroid tissue will require further studies to verify the tissue viability and functionality at different intervals. Regeneration and revascularization time for the heterotopic implant after a period of cryopreservation, are not yet established and will require further investigation addressing both subjects. Finally, a longer postoperative period is also necessary to evaluate the outcome of thyroid function after transplantation.

The experimental study confirmed the viability and functionality of thyroid autotransplantation implanted in skeletal muscles with evidence of cell proliferation and without cellular apoptosis. This surgical strategy was effective in the treatment of postoperative hypothyroidism.

## Data Availability Statement

The raw data supporting the conclusions of this article will be made available by the authors, without undue reservation.

## Ethics Statement

The animal study was reviewed and approved by Ethics Committee on the Use of Animals of the Federal University of Rio de Janeiro.

## Author Contributions

All authors have contributed to the study and are responsible for the contents: MV, WB-D, LM-A, SA-LS—technical procedures, acquisition, analysis, and interpretation of data. AC and OF—technical procedures. DD—critical revision. AS—intellectual conception and design of the study, analysis and interpretation of data, and critical revision. All authors contributed to the article and approved the submitted version.

## Funding

This study was supported by the Brazilian Council for Scientific and Technological Development (CNPq) (grant No. 304265/2018-7) and the Rio de Janeiro State Foundation for Research Support (FAPERJ) (grant No. E-26/202.921/2019).

## Conflict of Interest

The authors declare that the research was conducted in the absence of any commercial or financial relationships that could be construed as a potential conflict of interest.
